# Graduate grade inflation at a U.S. research-intensive university: A 22-year longitudinal analysis

**DOI:** 10.1371/journal.pone.0341315

**Published:** 2026-03-25

**Authors:** Vivien Lee, Nathan R. Kuncel, Paul R. Sackett

**Affiliations:** University of Minnesota – Twin Cities, Minneapolis, Minnesota, United States of America; University of Granada: Universidad de Granada, SPAIN

## Abstract

The phenomenon of grade inflation has been studied extensively at high school and undergraduate levels, yet little is known about its occurrence in graduate education. This study bridges this gap by examining graduate grade inflation using data from one U.S. research intensive university, covering two decades of admissions across 75 master’s programs (*N* = 24,815) and 78 doctoral programs (*N* = 15,701). Relying on both linear and ordinal multilevel models, we investigated the presence of grade inflation and potential variations by degree level and individual academic programs at the program level. Our findings provide preliminary evidence for the presence of graduate grade inflation and suggest that the magnitudes differ across individual academic programs. There is also evidence showing that the trend of grade inflation significantly differed across master’s and doctoral programs. This apparent inflation undermines the signaling value of grades for employers and admission decisions in the labor market and academic selection, as well as for research, feedback, and learning purposes. Future research should replicate our findings using multi-institutional samples and examine drivers of graduate grade inflation to more accurately estimate its magnitude.

## Introduction

In recent decades, the phenomenon of grade inflation has received widespread attention across the world [e.g., 1–3]. Grade inflation can be defined as increases in course grades that are not attributable to concurrent increases in the quality of students’ work or student ability [4,5], and typically manifests as an increase in GPAs or a shift in grade distribution, where top grades become more common, leading to a compression at the upper end of the grading scale. For instance, in the U.S., researchers have found that universities show an increasing trend of average GPA across time and that the “A” is now the most common grade among college students in the U.S. [3,6]. Similarly, Bachan [1] reported that, even after controlling for student ability, there is still evidence for grade inflation at undergraduate level in the UK such that the proportion of students gaining first class and upper second-class honor degrees increased over time.

Importantly, although researchers have sometimes equated the observed grade increases with grade inflation, observed grade increases are only a prerequisite for grade inflation. Observed grade increases are a necessary yet insufficient condition for true grade inflation as observed grade improvements could be attributed to explanations other than changing student characteristics that may not correspond to a concurrent increase in students’ quality of work [e.g., 5,7]. Existing research on grade inflation in undergraduate education has identified three major categories of factors that could lead to observed grade increases: institutional/department, instructor, and student characteristics. For example, institutions and/or departments may implement grading policies to attract students in less popular fields or deter students in more popular fields in order to stabilize enrollment [8]. The increasingly commercialized nature of higher education in recent years may also impact instructor characteristics such as leniency bias when it comes to assessing students. Berezvai et al. [9] found that assigning students better grades was associated with more positive student evaluations of teaching in two different universities in Central Europe. More recently, using a sample of Korean university students, Park and Cho [10] discovered that when students received grades lower than expected, they might retaliate by providing lower evaluation ratings. Given that student evaluations of teaching can have substantial impact on faculty promotion and tenure decisions [5,11], instructors may choose to be lenient and assign better grades out of concerns for their career, contributing to the observed grade increases. Indeed, Matos-Díaz [12] showed that grade incentives can be powerful in raising students’ expected grades and increasing students’ willingness to take another course with the same instructor, which are likely to be linked to higher student evaluations of teaching, in a sample of Puerto Rican students. Simultaneously, the cost-cutting trend of hiring more part-time adjunct faculty, who often face even more pressure to keep students happy and satisfied, may also exacerbate such bias [13]. Finally, changes in student characteristics such as ability, demographics, course-taking pattern, and effort also play a significant role in explaining observed grade increases. However, these factors are typically not considered determinants of grade inflation as they likely correspond to genuine changes in the quality of student work [5]. Grades are not inflated if they reflect an improvement in learning and the quality of assignments. Thus, controlling for explanations like changing student qualities and factors other than changing student characteristics will be crucial in detecting the presence of grade inflation and accurately estimating its magnitude as well as distinguishing it from legitimate grade improvement.

Indeed, a number of studies have documented grade inflation/observed grade increases over time and examined the factors contributing to this trend across educational levels and countries. For instance, using a nationally representative sample of Portuguese secondary school students from 2010–2019, Silva et al. [14] revealed evidence of rising grades, particularly among private schools compared to public schools. Similar findings have also been observed among high school students in the United States [15]. To better understand drivers underlying grade inflation in secondary education, Arrafii [**16**] investigated grading practices in Indonesia and identified both academic and non-academic influences on teachers’ grading decisions, including students’ effort, participation, and extracurricular involvement. Comparable trends have also been observed at the undergraduate level [e.g., 1,3,6], where grade inflation appears across both introductory and advanced courses [17]. Concerned with the phenomenon of grade inflation, Ullah et al. [18] conducted in-depth interviews with faculty members in Pakistan and found that institutional pressures, such as maintaining enrollment, accommodating underprepared students, and improving teaching evaluations, were key contributors to grade inflation. Collectively, these findings suggest that grade inflation is a pervasive global phenomenon, which has prompted educators and researchers to come up with ways to combat grade inflation [19,20].

Despite extensive research at high school and undergraduate levels [3,6,17,21], grade inflation remains a relatively underexplored phenomenon in graduate education. A report published by the UNESCO in 2024 [22] found that global enrollment in tertiary education in continents such as North America, Europe, Latin America, Africa, and Asia has more than doubled from 2000–2022. In the United States, the number of master’s and doctoral degrees awarded increased by 16 percent and 20 percent, respectively, from years 2011–2012 to 2021–2022 [23]. This similar pattern is observed in Europe where there is a steady increase in the proportion of people attaining tertiary education, which include master’s and doctoral graduates, in recent years [24]. Given that the number of graduate degrees awarded is on the rise across the world, it becomes increasingly crucial to assess the magnitude of grade inflation at a graduate level in order to better gauge the utility of school grades to schools, employers, and students over time. Graduate grade inflation negatively affects our ability to evaluate admission assessments, conduct research on graduate school learning, and make use of graduate school grades for administrative purposes like awards and fellowships. To this end, our study has two primary goals: First, using US-based data from 75 master’s programs (*N* = 24,815) and 78 doctoral programs (*N =* 15,701) within a single institution, we explored the presence of graduate grade inflation at the individual program level across 22 years while controlling for student prior ability. Second, in line with existing literature on undergraduate grade inflation, we investigated potential differences in the magnitudes of graduate grade inflation by exploring differences by program type and degree level. It is our hope that this study could fill a critical gap in the literature and stimulate more research on grade inflation at a graduate level. Understanding whether grade inflation exists at a graduate level is a first step toward addressing other questions asked at secondary and undergraduate levels.

### Grade inflation: why should we care?

Signaling theory [25] suggests that because underlying characteristics are difficult to observe directly, individuals rely on signals to guide important decisions such as hiring and school admission. In the educational context, academic achievement (i.e., GPA) serves as one such signal. Beyond reflecting persistence and mastery across a variety of subjects, grades provide an observable indicator of student quality to schools and employers and serve as feedback to students about their own learning progress. However, grade increases not tied to genuine improvements in student performance create situations where high-performing and mediocre students receive indistinguishable grades (e.g., 4.0), rendering school grades useless as an information source and making it difficult to differentiate between them. In this sense, grade inflation erodes the external screening function of grades for prospective employers and admission officers [26], while also weakening their internal function on providing feedback on student progress.

### Signaling power to schools and employers: student quality

Understanding the phenomenon of grade inflation is crucial for helping schools and employers gain a better understanding of the power of school grades in signaling student quality for school admissions or when they enter the workforce [27]. At school, school grades are frequently used for making decisions regarding opportunities like merit-based scholarships and fellowships alongside other criteria. Similarly, graduate school grades in a master’s program are often considered for admissions into a doctoral program. Students performing well academically will have a higher chance of getting rewards. The inherent reduction in variability in school grades that comes with grade inflation may make it more difficult to differentiate students. Over time, this may render school grades less useful for making such decisions.

In the workplace, despite doubts surrounding the value of school grades in helping organizations hire talent [28], receiving excellent school grades is undoubtedly a symbol of students’ continuous efforts and persistence in performing well in their courses, which is often tied to conscientiousness, a personality trait that individuals bring to their job that could significantly impact job-related outcomes like counterproductive work behavior and on-the-job performance [29,30]. Indeed, recent meta-analytic evidence also confirmed the predictive validity of academic performance—at high school, undergraduate as well as graduate levels—for predicting job performance [31]. Thus, graduate grade inflation may compromise the criterion-related validity of school grades for predicting future on-the-job outcomes, making it increasingly difficult to distinguish between good and exceptional performers both in educational and occupational settings over time. Grade inflation at individual institutions may also undermine a university’s reputation in the eyes of prospective students and employers.

### Signaling power to students: learning and degree progress

Grade inflation must reduce the effectiveness of school grades as feedback to students as it runs the risk of miscommunicating performance standards [20]. Grades often serve as a feedback tool when individualized feedback from instructors is not feasible [11,32]. They have the potential to influence student performance, motivation as well as learning strategies. Koenka et al. [33] conducted a meta-analysis on the impact of grades in K-12 education; they found that students who received grades performed better than students who did not receive grades but at the same time displayed lower academic motivation, providing support for the notion that school grades play a role in influencing student performance and motivation.

Primary studies also confirmed the impact of school grades on performance and motivation. For instance, Main and Ost [34] found that course grades on a prior exam predicted student performance on a subsequent exam. Similarly, Gray and Bunte [35] found that low grades are tied to increased subsequent course performance. These findings are not surprising as students may change their self-regulatory strategies and reallocate their resources toward learning based on the grades (i.e., feedback) they receive [36].

Grades could also prompt students to seek additional information about their performance and improve their work or learning. There is some evidence suggesting that feedback seeking mediates the relationship between conscientiousness and final grades [37]. Accordingly, if grade inflation reduces the meaningfulness of school grades and creates a scenario where everything yields an A, its utility as feedback to students will also decline. After all, there is little reason to seek additional feedback or alter performance or learning strategies if they make little to no difference to their grade. Critically, when instructor-assigned grades provide limited signal to students, alternative evaluation methods such as structured peer assessment where students provide and receive feedback, may be considered. In a group of graduate students, Landry et al. [38] found that peer assessments can yield grading comparable to instructors and that feedback improved assignment quality. Meta-analytic evidence supports the notion that peer assessments are generally effective in improving academic performance [39]. Furthermore, narrative feedback focused on describing how students currently do and how they can improve might also be used in lieu of traditional numeric grade to better support student learning and performance [40].

### Other reasons for why school grades matter

In addition to the utility of school grades in predicting important outcomes and providing feedback on student learning, school grades are often used as a measure of learning and academic performance**—**constructs of interest to many. Because grade inflation corresponds to an increasingly restricted range of school grades and that the correlation between two variables is a function of their variability [41], the phenomenon of grade inflation might also paint a misleading picture of the criterion-related validity of other predictors used in graduate school admission such as the GRE scores. In other words, since grades are often used to measure learning as an outcome, to the extent that school grades are range restricted, the observed criterion-related validity of their predictors will be attenuated and distorted.

### Hypothesis and research question development

#### Detecting the presence of graduate grade Inflation.

Grade inflation at a graduate level is arguably more complex than at an undergraduate level because of more extreme restriction of talent and different attitudes towards grades among graduate instructors. On the one hand, grade inflation may not be present in graduate programs because the admissions process tends to be more selective; competitive programs often have stricter standards than undergraduate admission and underqualified applicants may self-select out of applying. This implies a greater degree of range restriction on ability and academic performance in graduate education, leaving less room for average grades to grow over time. On the other hand, grade inflation could still occur as instructors may be more inclined to award higher grades to graduate students than to undergraduates.

At an undergraduate level, school grades play an instrumental role in signaling student quality to graduate school admission offices as well as recruiters for entry level positions, which is not always the case at the graduate level. Knowing that differentiating students may not carry as much weight as they do in undergraduate education, instructors may be more less likely to adopt a criterion-referenced or self-referenced grading orientation, where students are evaluated against an absolute grading standard or against their past self, than norm-referenced grading orientation, where students are evaluated relative to other students in a course [11,42]. This means that a greater number of A’s may be awarded to graduate students compared to undergraduate students. As mentioned, because observed grade increases could be attributed to reasons other than increases in student quality and may not mean grade inflation, we controlled for admitted students’ baseline ability as indexed by GRE scores as a potential explanation for observed grade increases. If grade increases are still observed after controlling for prior ability, then observed grade increases is unlikely to be due to changes in student ability, providing evidence for grade inflation.

***Research question 1.*** Is there evidence for *graduate* grade inflation, as shown by consistent increasing trend of school grades over time even after controlling for program level baseline ability?

### Potential differences in the magnitudes of graduate grade inflation

***Academic Programs across Disciplines*.** The magnitude of grade inflation may differ by the type of program due to potential disciplinary differences in rigor and grading philosophies endorsed by faculty. The idea that there are interdisciplinary differences in the magnitude of grade inflation has been extensively studied and detected at an undergraduate level (e.g., 11). For instance, using a sample from University of Michigan, Achen and Courant [8] explored potential differences in the grades by department and found that, on average, instructors of courses in fields such as math and chemistry tended to record lower grades than those in English. One explanation might be objectivity in scoring where science and math courses tend to have assignments and quizzes with right or wrong answers that are graded objectively, whereas humanities courses have more subjective evaluation. Similarly, Rojstaczer and Healy [3] examined the magnitude of grade inflation using a sample from 119 institutions and found that schools with a science and engineering focus, where students tend to take more STEM courses, are less likely to award high grades (i.e., A’s and B’s) compared to other schools that are equally selective.

However, as discussed, grading in graduate programs often emphasizes research skills, critical thinking, and scientific contributions, which may result in more subjective, interpretation-based grading practices even within STEM disciplines. Furthermore, arguments about controlling class sizes and maintaining enrollment that are often used to explain stricter grading in STEM fields at the undergraduate level may be less relevant in graduate programs, where smaller and more specialized classes are the norm. Therefore, it remains unclear whether similar interdisciplinary grading differences exist at a graduate level.

***Research question 2****.* Does the magnitude of graduate grade inflation differ across academic programs?

***Master’s versus Doctoral Programs*.** Aside from disciplinary differences, the magnitude of grade inflation may differ by degree level. The phenomenon of graduate grade inflation is inherently more complex than undergraduate grade inflation because there is more than one degree level within graduate programs, including master’s and doctoral, each with its own selection standards, admission objectives, course content, and grading policies. These distinctions can contribute to varying magnitudes of grade inflation by degree level. Grade inflation may be more pronounced in master’s than doctoral programs for two primary reasons: First, because doctoral programs are generally more selective than master’s programs [43], one would reasonably expect students’ academic performance to be more range restricted to begin with [41], thereby leaving less room for growth over time. Second, the leniency bias in grading might be less common in doctoral programs because funding and financial assistance are more readily available at a doctoral level [44], which could alleviate instructors’ pressure to please students or attract enrollment that is common in self-funded master’s programs.

Alternatively, one could argue that grade inflation may be stronger in doctoral than master’s programs due to differences in curriculum design and perceived importance of school grades. Whereas master’s coursework is more likely to be lecture-based where class sizes tend to be larger, doctoral courses tend to be discussion-based with smaller class sizes and a focus on developing critical thinking and research skills so that students can contribute to the body of scientific literature in their respective fields [45]. These differences in course size and instructional methods may manifest as differences in school grades. For example, Ekstrom et al. [11] found that class size is inversely correlated with students’ grades and that faculty tend to use different instructional methods for introductory compared to advanced courses. Furthermore, the perceived value of school grades may also differ for master’s and doctoral graduates. Grades often matter less to employers and students at doctoral level as students are generally valued more for their domain-specific knowledge and specific accomplishments like publications than their school grades, and doctoral students face less pressure when it comes to education advancement. Hence, instructors may feel less pressure to use normative grading as its implications for future decision making are minimal. Taken together, it is unclear whether grade inflation would be stronger at a master’s or doctoral level.

***Research question 3.*** Is the magnitude of grade inflation significantly different for master’s versus doctoral programs?

### The current project

The current study aims at developing a deeper understanding of the phenomenon of grade inflation at a graduate level. Using a large dataset on 75 master’s programs (*N* = 24,815) and 78 doctoral programs (*N* = 15,701) covering over two decades of graduate admissions at a single university, we leverage multilevel modelling (MLM) and investigate the presence of grade inflation in graduate education at the level of the individual academic program as well as the extent to which observed grade increases at the academic program level is attributable to actual grade inflation. Our study has substantial contributions. First, we fill a critical gap in the literature by investigating the presence of grade inflation in graduate education. Second, by exploring graduate grade inflation, our findings provide important information to educators and employers on the meaning and utility of school grades at a graduate level.

## Method

The study was approved by the institutional review board at the University of Minnesota (IRB#STUDY00024904) with a waiver of informed consent.

### Sample

A sample of *N* = 44,678 graduate students from at one large US Midwestern University admitted across 24 years from years 1999–2022 was included in the initial dataset. The data, which were anonymized in advance, were shared with the research team by the graduate school in August 2023. Data were accessed for the current project on October 23, 2023. Students were enrolled in 105 master’s programs and 90 doctoral programs. All students had already completed their final exams, if at a master’s level, and preliminary exams, if at a doctoral level. Due to the nature of the analysis, students admitted from 2021–2022 were excluded from the analyses as the outcome criterion was unavailable for most students. Additionally, we further coded and removed programs where data were too sparse due to missing data for many time points. This resulted in a final sample of *N =* 24,815 students from 75 master’s programs and *N =* 15,701 from 78 doctoral programs.

### Measures

***Classification of instructional program (CIP) codes*.** Our grouping variable was academic programs, which were recoded to be consistent with CIP, a taxonomy of academic titles developed by the U.S. Department of Education [46]. Examples of CIP programs include research and experimental psychology, educational psychology, history, physics, and geography. The full list of CIP programs available in the current study is included in Tables S1a and S1b in S1 File in the supporting materials.

***Graduate Cumulative Grade Point Average (GPA).*** Individual graduate school performance was operationalized as their cumulative GPA after they have completed all required coursework for their program. GPA information was extracted from university records.

***Graduate Record Examination (GRE).*** To control for students’ ability upon entering graduate school as a potential reason for observed grade increases, students’ GRE total scores (i.e., sum of verbal and quantitative component scores) were used as a level 1, student-level covariate. Because the scale of GRE changed in year 2011, old scores (out of 800 per component) before 2011 were transformed into the new scale (out of 170 per component) based on a concordance chart by Educational Testing Service, which can be found as part of our supplementary materials. Because missing GRE scores are likely policy-driven, due to GRE being optional in some academic programs and policy changes during the Covid-19 pandemic, and not missing at random or missing completely at random, a missingness indicator was created to distinguish cases with and without missingness [47]. Missing values for the GRE were imputed with the value “0”, which corresponds to the grand mean after centering to aid interpretation.

***Degree Level.*** This variable was coded based on the specific program students were enrolled in. 0 refers to master’s programs and 1 refers to doctoral programs.

***Sex.*** Students’ sex was dummy coded as 0 for female students and 1 for male students.

***Ethnicity.*** Ethnicity was coded as one of the following six groups: White, Black, Hispanic, Asian, Other, and Non-specified. White was used as the reference group.

### Analytic approach

To study the presence and magnitudes of graduate grade inflation across time, a series of eight linear mixed effects models where students (level 1) were nested within academic programs (level 2) was fitted. Time and GRE, including both GRE total scores and GRE missing indicators, were added as student-level covariates at level 1; degree level was input as a program-level covariate at level 2. Covariates were added in one at a time, allowing us to directly test our research questions. Additionally, although we considered a unified three-level model (time – program – degree level), two-level models were used because a unified model would contain only two level-3 units (i.e., master’s and doctoral) and that past simulation research has shown small sample size for higher level groupings may lead to inaccurate estimation of variance components as well as inferences [48]. Similarly, although other longitudinal approaches (e.g., fixed effects panel models) are viable, we employed multilevel modeling (i.e., linear mixed effects models) for both interpretability and alignment with our research questions [49]. Fixed effects models handle clustering by including higher-level grouping variables (in this case, academic programs) as dummy variables. With more than 150 master’s and doctoral programs in our dataset, this approach would yield over 100 dummy variables which could be cumbersome, whereas mixed effects models provide a more parsimonious and interpretable modeling framework by allowing the modeling of academic program as a random effect. Second, and more importantly, mixed effects models enable a direct examination of level 2 random effects, which is central to our research goals. RQ2 investigates the cross-level interaction between degree level (a level 2 variable) and time, and RQ3 evaluates random slopes for time across programs.

The assumptions of linearity, normality, homoskedasticity, and independence of observations were checked. Our baseline model was a random intercepts model with no predictor. Then, to estimate the average grade increase across time, time was entered as a predictor. Afterwards, a random intercepts-random slopes model, which assume differential growth rates in grades across academic programs, was fitted and compared to the random intercepts model on model fit. To explore potential non-linear effect of time, we fitted natural splines of time with varying degrees of freedom (*df*). A natural spline breaks the time predictor into segments and fits smooth cubic curves to each segment, joining them at knots. This flexibility allows us to test whether the effect of time departs from a simple linear trend. Following recommendations from Harrell [50], we started with a *df* of 3 up to 6 until there was no significant improvement in model fit as shown by $\chi^{\text{2}}$ likelihood ratio tests. Once the functional form of the relationship between time and GPA was determined, we controlled for individual-level GRE scores as a proxy of baseline ability to obtain more accurate estimates of grade inflation over time. A GRE missingness indicator was also included. Finally, degree level and the interaction between degree level and time were included to understand whether the magnitude of grade inflation differs for master’s versus doctoral programs. For all models, residual variance ($\alpha^{\text{2}}$) and R^2^ were reported to illustrate the amount of variance explained. Whereas Marginal R^2^ denotes the proportion of variance in the outcome that is explained by fixed effects only, conditional R^2^ denote the proportion of variance that is explained by both fixed effects and random effects.

To supplement our analyses, we conducted separate analyses for each degree level as a sensitivity check. In addition, because GPA values are skewed and clustered near the top of the scale, standard methods may give misleading results. Accordingly, we also fitted Bayesian multilevel ordinal models to confirm that the observed upward trend is real and not an artifact of model choice, given that individual grades are not continuous but grouped into ordered categories even though cumulative GPA form a continuous variable [51,52]. A Bayesian approach was selected over a frequentist approach because it is better suited for estimating complex models with many parameters, in this case natural spline terms and random effects, while producing more stable estimates. Specifically, GPA values were grouped into six approximately equal-sized categories exclusive of the upper bound (i.e., [-Inf,3.50], [3.50, 3.70], [3.70, 3.80], [3.80, 3.90], [3.90,4.00], [4.00, + Inf]). The assumption of proportion odds (PO) for time, which asserts that the effect of time is consistent across all GPA categories, was explicitly tested.

## Results

Student-level descriptive statistics for GPA and GRE by admission year and degree level are summarized in Table 1. The mean GPAs for both master’s and doctoral programs exhibit an increasing trend from 1999 to 2020 (*d*_masters_ = 0.56; *d*_doctoral_ = 0.36). For doctoral programs, there is a gradual but noticeable reduction in standard deviations of mean GPA over time. For GRE, there is also an increasing but weaker trend over time (*d*_masters_ = 0.33; *d*_doctoral_ = 0.53).

**Table 1:** Descriptive Statistics for GPA and GRE total scores by Admission Year at Student Level.

Admission Year	Master’s Level					Doctoral Level				
		GPA		GRE			GPA		GRE	
	*N*	*M*	*SD*	*M*	*SD*	*N*	*M*	*SD*	*M*	*SD*
1999	811	3.70	0.25	307.11	11.20	461	3.74	0.24	312.10	10.85
2000	1099	3.69	0.25	308.12	11.48	611	3.74	0.25	311.83	10.96
2001	1091	3.68	0.25	308.15	12.30	690	3.74	0.25	312.63	10.99
2002	1074	3.69	0.25	307.48	11.72	728	3.73	0.25	312.31	10.88
2003	1108	3.69	0.25	306.35	11.59	765	3.75	0.24	312.84	12.12
2004	1053	3.70	0.25	307.20	11.25	754	3.73	0.24	312.55	11.27
2005	1074	3.71	0.24	308.45	12.03	720	3.74	0.25	312.17	10.87
2006	1153	3.70	0.25	307.59	11.60	727	3.78	0.22	313.35	10.36
2007	1174	3.69	0.26	308.24	11.60	808	3.75	0.24	313.68	11.14
2008	1273	3.71	0.25	309.30	11.65	770	3.76	0.24	313.92	11.12
2009	1131	3.68	0.26	307.74	11.47	718	3.77	0.21	313.99	10.99
2010	1226	3.68	0.26	308.47	11.67	739	3.77	0.21	313.77	11.48
2011	1220	3.69	0.27	309.94	11.99	762	3.77	0.22	314.49	10.83
2012	1191	3.70	0.25	312.01	11.66	734	3.76	0.22	315.88	11.34
2013	1106	3.68	0.26	311.16	10.32	701	3.75	0.22	316.88	10.93
2014	1225	3.70	0.25	311.85	10.66	795	3.79	0.20	316.26	11.31
2015	1299	3.71	0.26	311.97	10.88	766	3.79	0.19	317.12	11.02
2016	1365	3.71	0.26	311.91	11.37	731	3.78	0.22	316.45	11.66
2017	1401	3.72	0.24	312.88	10.22	779	3.79	0.20	317.15	11.18
2018	1214	3.73	0.25	313.19	10.69	715	3.80	0.20	316.41	11.31
2019	1048	3.76	0.24	312.96	11.79	708	3.82	0.18	316.75	11.65
2020	479	3.82	0.20	311.13	12.80	519	3.82	0.19	318.02	11.38

*Note*. *N* = the number of student GPAs evaluated; *M* = the mean of variable; GPA is the Graduate school cumulative grade point average.

To test our assumptions, for normality, residuals at both level 1 and level 2 were inspected using QQ plots, showing evidence for normality. For homoskedasticity of variance, we examined fitted values versus residuals plots and found evidence violation of homoskedasticity. This is unsurprisingly as our dependent variable, GPA, is bounded between 0 and 4. Therefore, we utilized robust standard errors produced from sandwich estimator for our fixed effect estimates to ensure accurate inference. Finally, dependence of observations was confirmed by computing the intraclass correlation coefficient (ICC) based on the baseline model. Specifically, we estimated an ICC of 0.21, which indicates that 21% of variance in student GPA can be explained by the academic program to which they belong, providing strong justification for our modeling approach.

Overall, across the seven fitted models, marginal $R^{\text{2}}$ values ranged from .00 to .08 while conditional $R^{\text{2}}\;$values ranged from .21 to .34, indicating that both fixed and random effects accounted for a substantial proportion of the variance. Residual variance decreased steadily with the addition of predictors, suggesting improved model fit.

First, to answer our research question of whether there were significant grade increases over time (i.e., RQ1), a random intercepts-fixed slope model with only time as a predictor was fitted in Model 2. As shown in Table 2, time significantly predicted GPA at *p* < .001, suggesting the phenomenon of grade increases over time. Specifically, one year increase in time corresponds to 0.0049-point increase in GPA (*95% CI* = [0.0040, 0.0058]). It is worth noting that this increase is occurring with grades that are, on average, already very high compressing an already small variance.

**Table 2:** Results from Linear Mixed Effects Models.

	Model 1: Random Intercepts Model			Model 2: Random Intercepts Model with Linear Time Slope			Model 3: Random Intercepts-Random Slopes Model			Model 4: Random Intercepts-Random Slopes Model		
Fixed Effects	*b*	*95% CI*	*p*	*b*	*95% CI*	*p*	*b*	*95% CI*	*p*	*b*	*95% CI*	*p*
Intercept	3.7455	[3.7235,3.7675]	<.001	3.6925	[3.6666,3.7183]	<.001	3.6858	[3.6586,3.7129]	<.001	3.7010	[3.6744,3.7277]	<.001
Time	–	–	–	0.0049	[0.004,0.0058]	<.001	0.0054	[0.0045,0.0063]	<.001	–	–	–
ns(Time,4) 1										0.0409	[0.0176,0.0641]	<.001
ns(Time,4) 2										0.0387	[0.0163,0.0611]	.001
ns(Time,4) 3										0.1173	[0.0851,0.1496]	<.001
ns(Time,4) 4										0.1308	[0.1104,0.1513]	<.001
**Random Effects**												
$\sigma^{\text{2}}$	0.0481			0.0473			0.0469			0.0468		
Marginal R^2^/ Conditional R^2^	0.00/0.21			0.01/0.24			0.02/0.25			0.02/0.29		
**Model Comparison**				$\chi^{\text{2}}$(1) = 685.39, *p* <.001			$\chi^{\text{2}}$(2) = 197.57, *p* <.001			$\chi^{\text{2}}$(3) = 119.33, *p* <.001		
	**Model 5:** **Adding demographic variables**			**Model 6:** **Adding GRE as Level 1 covariate**			**Model 7:** **Adding Degree Level as** **Level 2 covariate**			**Model 8:** **Adding Degree Level x Time interaction**		
**Fixed Effects**	*b*	*95% CI*	*p*	*b*	*95% CI*	*p*	*b*	*95% CI*	*p*	*b*	*95% CI*	*p*
Intercept	3.7201	[3.6929,3.7473]	<.001	3.7378	[3.7089,3.7667]	<.001	3.7118	[3.6803,3.7433]	<.001	3.7071	[3.6727,3.7415]	<.001
ns(Time,4) 1	0.0431	[0.0206,0.0656]	<.001	0.0360	[0.0119,0.0602]	.005	0.0339	[0.0084,0.0593]	.011	0.0318	[-0.0028,0.0665]	.078
ns(Time,4) 2	0.0440	[0.0218,0.0663]	<.001	0.0253	[0.0029,0.0477]	.029	0.0276	[0.0052,0.0499]	.018	0.0288	[-0.0053,0.0629]	.104
ns(Time,4) 3	0.1255	[0.0926,0.1585]	<.001	0.1031	[0.0688,0.1373]	<.001	0.0997	[0.0638,0.1357]	<.001	0.1207	[0.0696,0.1718]	<.001
ns(Time,4) 4	0.1365	[0.1156,0.1575]	<.001	0.1216	[0.1012,0.1419]	<.001	0.1220	[0.1017,0.1424]	<.001	0.1372	[0.1083,0.1661]	<.001
Sex	−0.0166	[-0.0271,-0.0061]	.003	−0.0227	[-0.0336,-0.0117]	<.001	−0.0241	[-0.0345,-0.0136]	<.001	−0.0242	[-0.0346,-0.0137]	<.001
Ethnicity (Blacks)	−0.1713	[-0.1933,-0.1493]	<.001	−0.1396	[-0.1608,-0.1185]	<.001	−0.1411	[-0.1621,-0.1201]	<.001	−0.1411	[-0.1622,-0.12]	<.001
Ethnicity (Hispanics)	−0.0694	[-0.0869,-0.0519]	<.001	−0.0510	[-0.0686,-0.0334]	<.001	−0.0553	[-0.0727,-0.0379]	<.001	−0.0553	[-0.0726,-0.0381]	<.001
Ethnicity (Asians)	−0.0264	[-0.0453,-0.0074]	.010	−0.0344	[-0.0531,-0.0156]	<.001	−0.0380	[-0.0559,-0.0201]	<.001	−0.0380	[-0.0558,-0.0201]	<.001
Ethnicity (Others)	−0.1079	[-0.1328,-0.083]	<.001	−0.0835	[-0.1086,-0.0583]	<.001	−0.0850	[-0.1098,-0.0602]	.000	−0.0848	[-0.1097,-0.0598]	<.001
Ethnicity (Not Specified)	−0.0037	[-0.0264,0.0191]	.757	−0.0104	[-0.0315,0.0108]	.343	−0.0162	[-0.0352,0.0029]	.105	−0.0160	[-0.035,0.0031]	.108
GRE Missingness Indicator	–	–	–	−0.0116	[-0.0253,0.0022]	.108	−0.0103	[-0.0243,0.0036]	.153	−0.0107	[-0.0247,0.0033]	.141
GRE Total Score	–	–	–	0.0050	[0.0043,0.0056]	<.001	0.0048	[0.0041,0.0055]	.000	0.0048	[0.0041,0.0055]	<.001
Degree Level x ns(Time,4) 1	–	–	–	–	–	–	–	–	–	0.0067	[-0.0299,0.0434]	.720
Degree Level x ns(Time,4) 2	–	–	–	–	–	–	–	–	–	−0.0038	[-0.0415,0.034]	.846
Degree Level x ns(Time,4) 3	–	–	–	–	–	–	–	–	–	−0.0506	[-0.1045,0.0032]	.072
Degree Level x ns(Time,4) 4	–	–	–	–	–	–	–	–	–	−0.0309	[-0.0677,0.0059]	.107
**Random Effects**												
$\sigma^{\text{2}}$	0.0457			0.0439			0.0435			0.043453		
Marginal R^2^/ Conditional R^2^	0.04/0.30			0.07/0.33			0.08/0.34			0.08/0.34		
**Model Comparison**	$\chi^{\text{2}}$(6) = 985.39, *p* <.001			$\chi^{\text{2}}$(2) = 1617.10, *p* <.001			$\chi^{\text{2}}$(1) = 356.75, *p* <.001			$\chi^{\text{2}}$(4) = 20.55, *p* <.001		

*Notes. N* = 40,516; ns stands for natural splines which were used to model the non-linear effects of time; GRE total score was mean-centered to facilitate intercept interpretation; reference group for Ethnicity is Whites and reference group for Sex is Female; missingness indicator (0 = not missing; 1 = missing); degree level (0 = masters; 1 = PhD)

Second, to test our RQ2 which concerns whether the extent of grade increases significantly differs across academic programs, we compared the model fit of the random intercepts-fixed slope model to that of the random intercepts-random slopes model using likelihood ratio tests (i.e., Model 3). As shown in Table 2, results from $\chi^{\text{2}}$likelihood ratio test revealed that the random intercepts-random slopes models demonstrate a significantly better fit ($\chi^{\text{2}}$[2] = 197.57, *p* < .001), providing evidence that the rate of grade changes differs across programs.

To confirm the linearity of the effect of time, we fitted the random intercepts-random slopes model with natural splines with degrees of freedom ranging from 3 to 6. A series of model comparisons using likelihood ratio tests revealed that the model with 4 degrees of freedom emerged as the best fitting model (i.e., Model 4), which was used as the basis for later models. Four degrees of freedom mean that there are up to four segments and three inflection points for the relationship between time and GPA.

Third, to account for student demographic characteristics as a potential explanation for observed grade increases, individual-level sex and ethnicity were entered as level 1 covariates in Model 5. Both sex and ethnicity significantly predicted GPA such that students who were male and students who identified as Black or Hispanic showed a smaller increase in GPA.

Fourth, to rule out increased students’ baseline ability as an alternative explanation for observed grade increases, individual-level GRE total scores and the GRE missingness indicator were added as level 1 covariates in Model 6. To make intercept estimates more interpretable, GRE was grand mean centered by subtracting the grand mean of GRE scores across all programs and all time points. Unsurprisingly, as shown in Table 2, GRE scores significantly predicted GPA such that a one-point increase in GRE above the grand mean predicted 0.0050-point increase in GPA. GRE missingness indicator was not statistically significant, indicating that cumulative GPA did not differ significantly for those with and without GRE scores. Regarding the effect of time, results continued to show that time significantly and nonlinearly predicted GPA even after controlling for GRE scores, indicating potential grade inflation.

**Fig 1:**
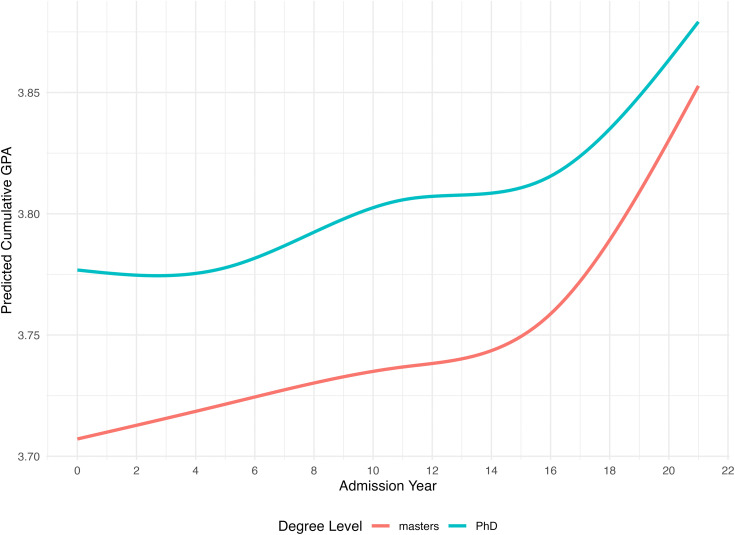
Predicted GPA by year and by degree level. Predicted values are conditional on model covariates and reflect the reference groups used in the analyses (female and White students), with GRE held at its mean (GRE = 0) and the missing GRE indicator set to 0.

Finally, to explore whether the rate of grade inflation differs for master’s versus doctoral programs (i.e., RQ3), we input degree level as a level 2 covariate as well as the interactions between degree level and time in two steps. The first model has only the main effect of degree level (i.e., Model 7) and the second model has both the main effect of degree level and the interactive effects between degree level and time (i.e., Model 8). Results are summarized in Table 2. In Step 1, we found a significant effect for degree level at *p* < .001 such that students enrolled in doctoral programs have on average 0.06 points higher in cumulative GPA compared to students enrolled in master’s programs. In Step 2, we compared two nested models using $\chi^{\text{2}}$ likelihood ratio test. Results showed that the model with the interactive effects had significantly better fit ($\chi^{\text{2}}$ [4] = 20.55, *p* < .001), providing support for the idea that the magnitude of grade inflation varies by degree level. Importantly, this is not in conflict with the nonsignificant coefficients for individual interaction terms reported. It is because natural splines decompose time into multiple basis functions, each term captures only a portion of the overall trajectory. As a result, the significance of the interaction must be evaluated jointly across all spline terms, rather than from any single coefficient. The overall finding suggests that the trend of grade inflation is non-linear and that this trend is statistically different for master’s and doctoral programs. To better illustrate the differences by degree level, Fig 1 shows the non-linear trend of predicted GPA by degree level conditional on student characteristics. For master’s programs, GPA shows a steady upward trend in the earlier years, followed by a period of (relative) stability, and a marked increase in recent years. For doctoral programs, GPA remained relatively flat initially, then rises modestly before displaying a shaper increase in the most recent admission years. These patterns suggest that grade inflation has accelerated in both programs, though it appears to unfold differently across degree levels.

Follow-up analyses using linear mixed effects models separating master’s and doctoral programs confirm the observed non-linear effects of time.

Results from a series of Bayesian multilevel ordinal models, including one overall model and two degree-specific models, also indicated the presence of grade inflation, suggesting that the upward trend in GPA over time was observed even with an alternative model choice. There was no evidence for the violation of the proportional-odds assumption in the overall and master’s level models, whereas the doctoral level model showed mild evidence of a potential violation, suggesting that the magnitude of grade inflation may not be uniform across GPA categories. Because time was modeled using natural splines, its effect cannot be summarized by a single regression coefficient. Thus, we summarized the effect of time by comparing the model’s predicted log-odds of being in a higher GPA category at two time points (1999 and 2020) and exponentiating this difference to obtain an odds ratio. The odds ratio represents the change in the cumulative odds of being in a higher GPA category between these two admission years while holding other covariates constant. Consistent with the linear models, results from the overall Bayesian multilevel ordinal model indicated higher GPA categories over time; specifically, the cumulative odds of being in a higher GPA category were 43% greater for students admitted in 2020 compared to those admitted in 1999 (*OR* = 1.43, *95% Crl* = [1.28, 1.60]). This general upward trend was similarly observed in the degree-specific models. Moreover, the ordinal models also provided evidence that the magnitude of grade inflation likely differed across degree levels. Detailed results from the Bayesian multilevel ordinal models are provided in Tables S2-S4 in S1 File in the supporting materials.

## Discussion

Our study demonstrated that grade increases are observed over time at a graduate level for both master’s and doctoral programs even after controlling for student sex, ethnicity, and prior ability, providing preliminary evidence for graduate grade inflation. Our findings showed that graduate grade inflation is nonlinear, rather than linear, with a sharp increase among students admitted years between 2017 and 2020. This is likely due to grading and instructional changes during the Covid-19 pandemic. As cumulative GPA was typically recorded at the end of year 2 among master’s students and year 3 among doctoral students, grades of students admitted around this period will be partially impacted and are likely to be inflated, as noted in some institutions (e.g., [53]). Importantly, there is still a notable grade increase between years 1999 and 2017 (*d*_masters_ = 0.10; *d*_doctoral_ = 0.22).

**Fig 2:**
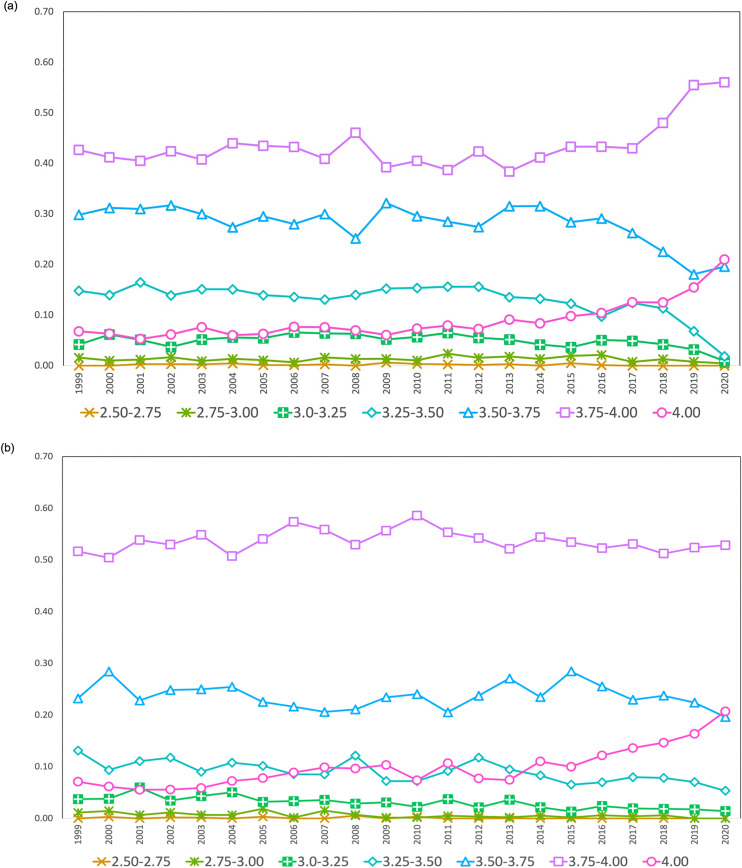
Proportions of students belonging to different grade buckets by admission year at (a) master’s level and (b) doctoral level. All buckets are exclusive of upper bound.

In terms of degree level comparison, we found that the cumulative magnitude of grade inflation is slightly higher for master’s programs than doctoral programs between 1999 and 2020, presumably due to potential ceiling effects among doctoral programs, which have higher starting mean academic performance in our sample. Indeed, individual intercepts and slopes tend to be negatively correlated such that programs with higher starting intercepts (i.e., mean GPA in year 1999) tend to show lesser growth over time (*r* = −.48). To gain further insights into the differences between master’s and doctoral programs, we explored grade distributions across time at both degree levels descriptively. As shown in Fig 2, both degree levels saw an overall increase in the proportion of students obtaining a 4.0, the maximum grade possible, in recent years. However, there is also a noticeable increase in the proportion of students obtaining 3.75–4.00 and visible decrease in the ranges of 3.25–3.50 and 3.50–3.75 for master’s level program, a pattern that is not seen for doctoral level programs, revealing distinct changes in grade distribution between master’s and doctoral programs.

**Fig 3:**
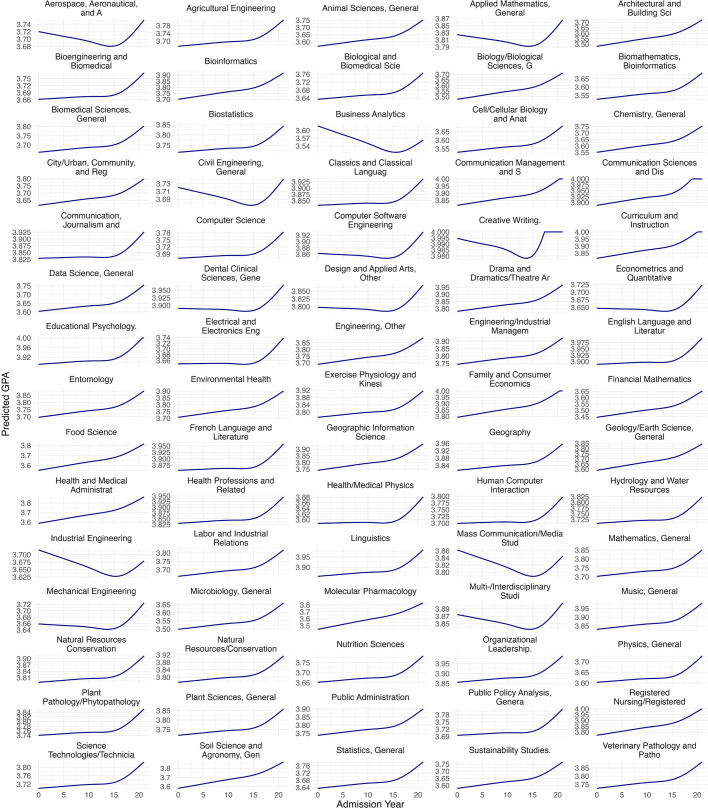
Program-specific GPA trajectories at master’s level. Predicted values are conditional on model covariates and reflect the reference groups used in the analyses (female and White students), with GRE held at its mean (GRE = 0) and the missing GRE indicator set to 0.

**Fig 4:**
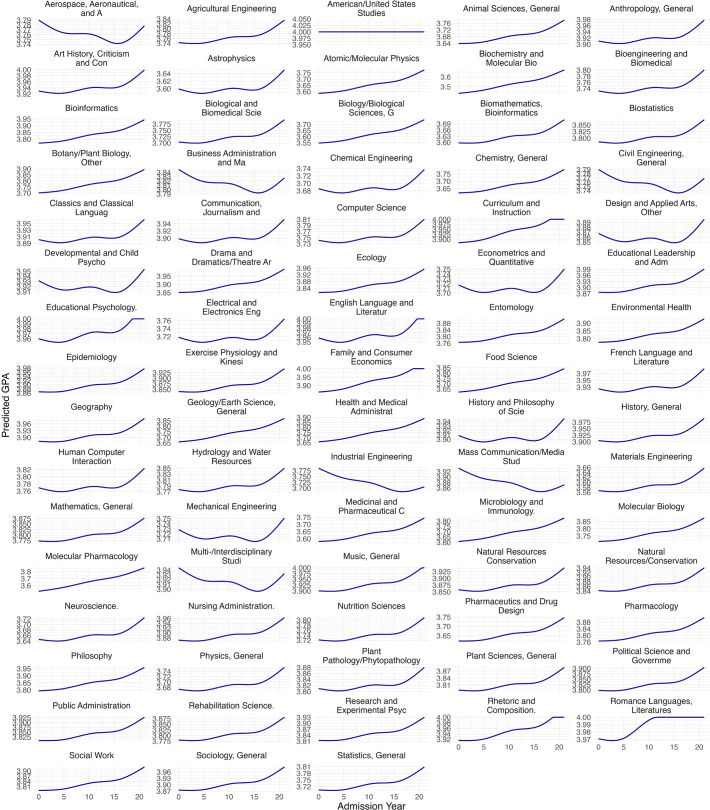
Program-specific GPA trajectories at doctoral level. Predicted values are conditional on model covariates and reflect the reference groups used in the analyses (female and White students), with GRE held at its mean (GRE = 0) and the missing GRE indicator set to 0.

In addition, as shown in Figs 3 and 4, the trajectory of grade inflation varied substantially across academic programs. While nearly all programs exhibited increases between 2017 and 2020, patterns from 1999 to 2016 were more heterogeneous. In fact, several programs, such as aerospace engineering, industrial engineering, multidisciplinary studies, and media studies, displayed clear downward trends at both master’ and doctoral levels. These declines may reflect program-specific grading policies or philosophies, though our data do not allow us to direct examine or control for these mechanisms. This highlights the importance of considering program-level factors when examining grade inflation in future research. Moreover, contrary to the expectation that non-STEM programs might be more prone to inflation due to greater grading subjectivity, we did not observe systematic differences between STEM and non-STEM fields at either degree level.

### Study contributions

The current study has substantial contributions to both theory and practice. First, consistent with prior research on grade inflation at undergraduate level, our findings indicate that grade inflation likely occurs in graduate education, thereby bridging a crucial gap in the achievement and educational testing literature. Second, using MLM, our study also contributes to the literature by exploring the phenomenon of graduate grade inflation at a program level and producing program-level estimates of grade inflation when existing literature has tended to explore grade inflation either at an institutional level or student level. Third, to our knowledge, this is the first study to investigate the potential non-linear trends in grade inflation (while controlling for student ability), suggesting that the phenomenon of grade inflation may be more complex and nuanced than previously understood.

Additionally, this study provides practical and invaluable information to both employers and educators to aid decision making for selection in organizational and educational contexts. The existence of graduate grade inflation as evidenced by this upward trend of GPA, and increasingly compressed grade spread suggests that the ability of grades to signal differences in student academic achievement is impaired and that the meaning of grades is changing over time even at the graduate level [54,55]. Admission offices and employers might be concerned about the devaluation of graduate degree qualifications. Importantly, an increasingly compressed range of grades may compromise school grades’ criterion-related validity (and hence their utility) for predicting important outcomes such as future academic performance as well as job performance, thereby increasing the risk of admitting or hiring undesirable candidates, which can be costly to organizations [e.g., 43]. Finally, to the extent that grades are intended to give student accurate feedback about their performance, the results suggest that, increasingly, grading may be failing at that task. The average GPA was already an “A” average 25 years ago and it steadily compressed what was already a narrow range of GPAs. Educators might consider the utility of school grades as a feedback tool for students to reflect upon their progress in their program. To combat the issue of graduate grade inflation, educators might consider implementing standardized grading rubrics or offering coaching sessions to help faculty develop standardized assessment practices. As well, because instructors may be incentivized to give out higher school grades in exchange for more positive student evaluations, institutions should also rethink the use of student evaluations in tenure and promotion decisions.

### Limitations and future directions

However, the current project is not without limitations. First, the dataset utilized in the current project came from one institution, a large US Midwestern state university. Although the dataset is large and spans across more than twenty years, one may ask whether findings are idiosyncratic to the institution in question. We believe that our findings will very likely generalize to other institutions and other geographical regions for three reasons. First, the current study used a sample from a large university, where there were considerable changes in faculty and university leadership over the twenty-two-year timeframe, so it is unlikely that the observed grade increases are due to a single policy or a single group of faculty unique to the institution under study. Second, like most research-intensive universities, graduate students come from all over the world to study in a large number of disciplines and are likely to have varying characteristics, suggesting that this university’s candidate pool is broad, and does not reflect some set of local candidate pool features that make this an idiosyncratic sample. Third, grading is not centrally managed; faculty are largely free to determine their own grading standards, which should lead to differences in grading rather than a consistent trend of grade inflation. Together, these factors make it unlikely that graduate-grade inflation is merely a localized issue specific to the institution under study and instead suggest that it aligns with broader trends observed at the high school and undergraduate levels. In any case, we acknowledge that exploring this phenomenon with data from a single institution is limited and that the magnitude of grade inflation observed may not generalize fully to other institutions.

Future research should attempt to replicate the findings across institutions and geographical regions to confirm the presence and magnitude of graduate grade inflation at other institutions and test institution-level effects. If possible, replicating our findings using multi-institutional datasets will be optimal. If graduate grade inflation is consistently observed, students, educators, and prospective employers should re-evaluate the value of school grades in conveying meaningful information about student learning progress and overall student quality. New learning techniques like peer assessments, which is linked to learning, may be necessary for helping students reflect on their learning progress [56,57]. Instructors may also need to rely on formative assessments to gauge students’ level of learning. Likewise, in the workplace, employers may place less emphasis on school grades when distinguishing applicants and consider alternative measures of skills and competencies.

Second, we acknowledge that using GRE scores as a proxy for baseline student ability is imperfect. The GRE has undergone reforms, including changes in test format and scoring, and many universities—including the one examined in this study—have gradually adopted test-optional policies in recent years. Nevertheless, standardized test scores such as the GRE have long been regarded as a relatively direct and objective measures of cognitive ability in many fields such as Economics and Industrial/Organizational Psychology. This approach is also consistent with prior grade inflation research: for example, Kostal et al. [5] controlled for student-level SAT in their analysis, and Rojstaczer and Healy [6] used SAT scores as a proxy for institutional selectivity. Taken together, these examples demonstrate that the use of standardized test scores as proxies for student ability is well-established in the literature, thereby justifying our approach. At the same time, we encourage future researchers to triangulate multiple indicators of student ability whenever possible when studying grade inflation.

Third, while the current study provides evidence of graduate grade inflation, it was not possible to tease out factors leading to the observed grade increases. Only baseline ability, sex, and ethnicity were included and no other individual-level variables (e.g., undergraduate GPA) were included due to limited information retained in student records. Other additional sources of variance, including institutional/departmental, instructor, and student characteristics such as curricular changes and shifts in grading philosophy might also be potential alternative explanations for the observed grade increases (e.g., 39). However, there were no major policy reforms during this time with respect to grading. Given that the effect was observed in a wide number of programs, which are effectively isolated from each other with respect to grading philosophy, changes in grading policy is unlikely to fully explain the observed trend. To the extent that other indicators of student quality such as student effort and course taking patterns accounted for the observed increases over time, the magnitude of the observed grade inflation would be reduced. However, rather than providing a universal estimate of grade inflation, our intent is to provide preliminary evidence for grade inflation at a graduate level. We encourage future research to consider alternative explanations for the observed grade increases such as socioeconomic status, social support, effort, and motivation to learn to obtain a more precise estimate of the magnitude of graduate grade inflation. Additionally, to deepen our understanding of grade inflation in graduate education, future research should also explore the role of course design (e.g., curriculum content and class size), faculty characteristics (e.g., biases, composition, and grading philosophy), which prior studies have identified as potential mechanisms contributing to genuine grade inflation (e.g., 5). These factors can be examined quantitatively–by incorporating the above variables as controls—and qualitatively—through focus groups or in-depth interviews to understand the drivers of grade inflation from faculty and students’ perspectives.

Fourth, the current treatment of degree level may be over simplistic as there might be substantial differences among academic programs at the same degree level. For instance, thesis-based and practice-oriented master’s programs may have substantially different grading policies as differences in course content may necessitate different learning evaluation methods and students and course instructors may have different motivations. Future research should account for program type (i.e., research versus practice) as a predictor and examine if grade inflation is more readily observed in practice-oriented master’s programs. Similarly, the current study only examined grade inflation in research-oriented doctoral programs, professional doctoral programs such as medicine and dentistry were not included in our current analyses due to data sparsity across years. Future research could use multi-institutional samples with larger sample sizes to detect the presence and extent of grade inflation in these disciplines, which will be essential for developing an understanding of the utility of school grades in graduate education more broadly.

## Conclusion

To summarize, the current study addresses a critical gap in the literature by investigating the presence of graduate grade inflation at the program level. In parallel with prior research on undergraduate education, our results suggest that graduate grade inflation exists and that its extent varies across different academic programs. Grade inflation appears to be more pronounced at the master’s level than at the doctoral level. Our findings offer valuable insights to both employers and educators by demonstrating that the signaling power of graduate school grades in reflecting student quality may not remain constant over time.

## Supporting information

S1 File **Table S1a.** List of CIP master’s programs included in the current study. **Table S1B.** List of CIP doctoral programs included in the current study. **Table S2a.** Results from linear mixed-effects models for master’s programs. **Table S2b.** Results from linear mixed-effects Models for doctoral programs. **Table S3a.** Results from Bayesian multilevel ordinal models for master’s programs. **Table S3b.** Results from Bayesian multilevel ordinal models for doctoral programs. **Table S4. Results from Bayesian multilevel ordinal models for both degree levels.**
(ZIP)
