# Supplementary material for: Graduate grade inflation at a U.S. research-intensive university: A 22-year longitudinal analysis
Source: PLoS One. 2026 Mar 25;21(3):e0341315. doi: 10.1371/journal.pone.0341315 (PMC13016357; doi:10.1371/journal.pone.0341315)
Supplement: S1 File — Table S1a. List of CIP master’s programs included in the current study. Table S1B. List of CIP doctoral programs included in the current study. Table S2a. Results from linear mixed-effects models for master’s programs. Table S2b. Results from linear mixed-effects Models for doctoral programs. Table S3a. Results from Bayesian multilevel ordinal models for master’s programs. Table S3b. Results from Bayesian multilevel ordinal models for doctoral programs. Table S4. Results from Bayesian multilevel ordinal models for both degree levels. (ZIP) [file pone.0341315.s001.zip › Supporting Information/Supporting Information - Table S3a.docx]

| **Table S3a. Results from Bayesian multilevel ordinal models for master's programs** | | | | | | | |
| --- | --- | --- | --- | --- | --- | --- | --- |
|  |  | Model 1: Proportion Odds Model | | | Model 2: Partial Proportional Odds Model | | |
| **Predictor** | **Threshold** | **Estimate** | **95% CrI** | **Crl includes 0?** | **Estimate** | **95% CrI** | **Crl includes 0?** |
| **Fixed Effects** |  |  |  |  |  |  |  |
| Sex | All | -0.2818 | [-0.3316,-0.2299] | No | -0.2826 | [-0.3346,-0.2325] | No |
| Ethnicity (Blacks) | All | -1.2140 | [-1.3500,-1.0808] | No | -1.2120 | [-1.3459,-1.0791] | No |
| Ethnicity (Hispanics) | All | -0.4860 | [-0.6273,-0.3402] | No | -0.4866 | [-0.6262,-0.346] | No |
| Ethnicity (Asians) | All | -0.5127 | [-0.5806,-0.4447] | No | -0.5106 | [-0.5762,-0.4433] | No |
| Ethnicity (Others) | All | -0.7395 | [-0.9804,-0.5022] | No | -0.7380 | [-0.9878,-0.4799] | No |
| Ethnicity (Not Specified) | All | -0.3535 | [-0.4303,-0.2746] | No | -0.3534 | [-0.4323,-0.2732] | No |
| GRE Missingness Indicator | All | -0.1424 | [-0.2018,-0.0816] | No | -0.1416 | [-0.2031,-0.0809] | No |
| GRE Total Score | All | 0.0520 | [0.0493,0.0548] | No | 0.0520 | [0.0493,0.0548] | No |
| ns(Time,4) 1 | ≥ 3.5 vs < 3.5 | 0.2330 | [0.0628,0.3972] | No | 0.1387 | [-0.065,0.3371] | Yes |
| ns(Time,4) 1 | ≥ 3.7 vs < 3.7 |  |  |  | 0.2349 | [0.0495,0.4143] | No |
| ns(Time,4) 1 | ≥ 3.8 vs < 3.8 |  |  |  | 0.2840 | [0.1009,0.4613] | No |
| ns(Time,4) 1 | ≥ 3.9 vs < 3.9 |  |  |  | 0.2867 | [0.079,0.4804] | No |
| ns(Time,4) 1 | = 4.0 vs < 4.0 |  |  |  | 0.2446 | [-0.0414,0.5256] | Yes |
| ns(Time,4) 2 | ≥ 3.5 vs < 3.5 | 0.2076 | [0.0089,0.3984] | No | 0.2038 | [-0.0299,0.4334] | Yes |
| ns(Time,4) 2 | ≥ 3.7 vs < 3.7 |  |  |  | 0.1307 | [-0.0842,0.3307] | Yes |
| ns(Time,4) 2 | ≥ 3.8 vs < 3.8 |  |  |  | 0.1839 | [-0.0288,0.3912] | Yes |
| ns(Time,4) 2 | ≥ 3.9 vs < 3.9 |  |  |  | 0.2713 | [0.0457,0.4934] | No |
| ns(Time,4) 2 | = 4.0 vs < 4.0 |  |  |  | 0.3515 | [0.0782,0.6349] | No |
| ns(Time,4) 3 | ≥ 3.5 vs < 3.5 | 0.9292 | [0.5826,1.2708] | No | 0.9244 | [0.5176,1.3266] | No |
| ns(Time,4) 3 | ≥ 3.7 vs < 3.7 |  |  |  | 0.8487 | [0.4692,1.2195] | No |
| ns(Time,4) 3 | ≥ 3.8 vs < 3.8 |  |  |  | 0.9358 | [0.5570,1.3227] | No |
| ns(Time,4) 3 | ≥ 3.9 vs < 3.9 |  |  |  | 0.9598 | [0.5544,1.3636] | No |
| ns(Time,4) 3 | = 4.0 vs < 4.0 |  |  |  | 0.8042 | [0.2099,1.3815] | No |
| ns(Time,4) 4 | ≥ 3.5 vs < 3.5 | 1.2618 | [1.0257,1.488] | No | 1.0360 | [0.7555,1.3109] | No |
| ns(Time,4) 4 | ≥ 3.7 vs < 3.7 |  |  |  | 1.2143 | [0.9612,1.4596] | No |
| ns(Time,4) 4 | ≥ 3.8 vs < 3.8 |  |  |  | 1.2461 | [1.0011,1.4854] | No |
| ns(Time,4) 4 | ≥ 3.9 vs < 3.9 |  |  |  | 1.3926 | [1.1419,1.6355] | No |
| ns(Time,4) 4 | = 4.0 vs < 4.0 |  |  |  | 1.3791 | [1.0805,1.6642] | No |
|  |  |  |  |  |  |  |  |
| **Thresholds** |  |  |  |  |  |  |  |
| ≥ 3.5 vs < 3.5 | - | -1.9970 | - | - | -2.0091 | - | - |
| ≥ 3.7 vs < 3.7 | - | -0.6880 | - | - | -0.7172 | - | - |
| ≥ 3.8 vs < 3.8 | - | 0.0573 | - | - | 0.0713 | - | - |
| ≥ 3.9 vs < 3.9 | - | 1.0877 | - | - | 1.1166 | - | - |
| = 4.0 vs < 4.0 | - | 2.8068 | - | - | 2.7977 | - | - |
|  |  |  |  |  |  |  |  |
| **Model Comparison** |  |  |  |  |  |  |  |
| elpd **Δ** | 0.0000 | - | - | - | -3.3000 | - | - |
| se **Δ** | 0.0000 | - | - | - | 5.0000 | - | - |
| *Notes.* - As a sensitivity check, Bayesian multilevel ordinal regression models were fitted using the "brms" package in R. 4 chains are used with 2000 warm-up iterations, followed by 3000 iterations. A Bayesian approach as opposed to a frequentist approach was chosen as traditional, frequentist methods can struggle to produce stable results for complex models with many parameters estimated. - Estimate is the mean of the posterior distribution for a given parameter and they are expressed in log odds given that a cumulative logit link function was used.  - ns refers to natural splines which were used to model the non-linear effects of time - 95% Crl represents the 95% credibility interval which denotes that there is 95% probability that the true value of the parameter lies within the range.  - Model comparison was performed by comparing examining the leave-one-out cross-validation information criterion for the proportional odds model and the non-proportional odds model. Note that to ensure stable estimates and to prevent the model from being overparameterized, the assumption of proportion odds was only tested with time and not other predictors. elpd represents the difference in expected log predictive density (elpd) between the two models and se represents the difference in standard error. - Results did not show violation for the proportion odds assumption. | | | | | | | |
