# Supplementary material for: Graduate grade inflation at a U.S. research-intensive university: A 22-year longitudinal analysis
Source: PLoS One. 2026 Mar 25;21(3):e0341315. doi: 10.1371/journal.pone.0341315 (PMC13016357; doi:10.1371/journal.pone.0341315)
Supplement: S1 File — Table S1a. List of CIP master’s programs included in the current study. Table S1B. List of CIP doctoral programs included in the current study. Table S2a. Results from linear mixed-effects models for master’s programs. Table S2b. Results from linear mixed-effects Models for doctoral programs. Table S3a. Results from Bayesian multilevel ordinal models for master’s programs. Table S3b. Results from Bayesian multilevel ordinal models for doctoral programs. Table S4. Results from Bayesian multilevel ordinal models for both degree levels. (ZIP) [file pone.0341315.s001.zip › Supporting Information/Supporting information - Table S1a.docx]

**Table S1a. List of CIP master’s programs included in the current study**

| # | **CIP Program** |
| --- | --- |
| 1 | Aerospace, Aeronautical, and Astronautical/Space Engineering, General |
| 2 | Agricultural Engineering |
| 3 | Animal Sciences, General |
| 4 | Applied Mathematics, General |
| 5 | Architectural and Building Sciences/Technology |
| 6 | Bioengineering and Biomedical Engineering |
| 7 | Bioinformatics |
| 8 | Biological and Biomedical Sciences, Other |
| 9 | Biology/Biological Sciences, General |
| 10 | Biomathematics, Bioinformatics, and Computational Biology, Other |
| 11 | Biomedical Sciences, General |
| 12 | Biostatistics |
| 13 | Business Analytics |
| 14 | Cell/Cellular Biology and Anatomical Sciences, Other |
| 15 | Chemistry, General |
| 16 | City/Urban, Community, and Regional Planning |
| 17 | Civil Engineering, General |
| 18 | Classics and Classical Languages, Literatures, and Linguistics, General |
| 19 | Communication Management and Strategic Communications |
| 20 | Communication Sciences and Disorders, General |
| 21 | Communication, Journalism, and Related Programs, Other |
| 22 | Computer Science |
| 23 | Computer Software Engineering |
| 24 | Creative Writing |
| 25 | Curriculum and Instruction |
| 26 | Data Science, General |
| 27 | Dental Clinical Sciences, General |
| 28 | Design and Applied Arts, Other |
| 29 | Drama and Dramatics/Theatre Arts, General |
| 30 | Econometrics and Quantitative Economics |
| 31 | Educational Psychology |
| 32 | Electrical and Electronics Engineering |
| 33 | Engineering, Other |
| 34 | Engineering/Industrial Management |
| 35 | English Language and Literature, General |
| 36 | Entomology |
| 37 | Environmental Health |
| 38 | Exercise Physiology and Kinesiology |
| 39 | Family and Consumer Economics and Related Services, Other |
| 40 | Financial Mathematics |
| 41 | Food Science |
| 42 | French Language and Literature |
| 43 | Geographic Information Science and Cartography |
| 44 | Geography |
| 45 | Geology/Earth Science, General |
| 46 | Health and Medical Administrative Services, Other |
| 47 | Health Professions and Related Clinical Sciences, Other |
| 48 | Health/Medical Physics |
| 49 | Human Computer Interaction |
| 50 | Hydrology and Water Resources Science |
| 51 | Industrial Engineering |
| 52 | Labor and Industrial Relations |
| 53 | Linguistics |
| 54 | Mass Communication/Media Studies |
| 55 | Mathematics, General |
| 56 | Mechanical Engineering |
| 57 | Microbiology, General |
| 58 | Molecular Pharmacology |
| 59 | Multi-/Interdisciplinary Studies, Other |
| 60 | Music, General |
| 61 | Natural Resources Conservation and Research, Other |
| 62 | Natural Resources/Conservation, General |
| 63 | Nutrition Sciences |
| 64 | Organizational Leadership |
| 65 | Physics, General |
| 66 | Plant Pathology/Phytopathology |
| 67 | Plant Sciences, General |
| 68 | Public Administration |
| 69 | Public Policy Analysis, General |
| 70 | Registered Nursing/Registered Nurse |
| 71 | Science Technologies/Technicians, Other |
| 72 | Soil Science and Agronomy, General |
| 73 | Statistics, General |
| 74 | Sustainability Studies |
| 75 | Veterinary Pathology and Pathobiology |
