# Supplementary material for: Graduate grade inflation at a U.S. research-intensive university: A 22-year longitudinal analysis
Source: PLoS One. 2026 Mar 25;21(3):e0341315. doi: 10.1371/journal.pone.0341315 (PMC13016357; doi:10.1371/journal.pone.0341315)
Supplement: S1 File — Table S1a. List of CIP master’s programs included in the current study. Table S1B. List of CIP doctoral programs included in the current study. Table S2a. Results from linear mixed-effects models for master’s programs. Table S2b. Results from linear mixed-effects Models for doctoral programs. Table S3a. Results from Bayesian multilevel ordinal models for master’s programs. Table S3b. Results from Bayesian multilevel ordinal models for doctoral programs. Table S4. Results from Bayesian multilevel ordinal models for both degree levels. (ZIP) [file pone.0341315.s001.zip › Supporting Information/Supporting Information - Table S4.docx]

| **Table S4. Results from Bayesian multilevel ordinal models for both degree levels** | | | | | | | | | | |
| --- | --- | --- | --- | --- | --- | --- | --- | --- | --- | --- |
|  |  | Model 1:  Proportion Odds Model without interaction | | | Model 2:  Partial Proportional Odds Model without interaction | | | Model 3:  Proportion Odds Model with interaction | | |
| **Predictor** | **Threshold** | **Estimate** | **95% CrI** | **Crl includes 0?** | **Estimate** | **95% CrI** | **Crl includes 0?** | **Estimate** | **95% CrI** | **Crl includes 0?** |
| **Fixed Effects** |  |  |  |  |  |  |  |  |  |  |
| Sex | All | -0.2234 | [-0.2624,-0.1850] | No | -0.2238 | [-0.2630,-0.1846] | No | -0.2239 | [-0.2647,-0.1843] | No |
| Ethnicity (Blacks) | All | -1.1220 | [-1.2327,-1.0131] | No | -1.1190 | [-1.2270,-1.009] | No | -1.1229 | [-1.2325,-1.0174] | No |
| Ethnicity (Hispanics) | All | -0.4322 | [-0.5323,-0.3288] | No | -0.4307 | [-0.5339,-0.3235] | No | -0.4326 | [-0.5377,-0.3291] | No |
| Ethnicity (Asians) | All | -0.3933 | [-0.4408,-0.3442] | No | -0.3928 | [-0.4418,-0.3424] | No | -0.3929 | [-0.4428,-0.3434] | No |
| Ethnicity (Others) | All | -0.7903 | [-0.9683,-0.6087] | No | -0.7845 | [-0.9628,-0.5992] | No | -0.7879 | [-0.9747,-0.6033] | No |
| Ethnicity (Not Specified) | All | -0.1719 | [-0.2259,-0.1164] | No | -0.1720 | [-0.2276,-0.1152] | No | -0.1702 | [-0.2271,-0.1139] | No |
| GRE Missingness Indicator | All | -0.0866 | [-0.1322,-0.0387] | No | -0.0868 | [-0.1342,-0.0402] | No | -0.0906 | [-0.1366,-0.0435] | No |
| GRE Total Score | All | 0.0429 | [0.0409,0.0451] | No | 0.0429 | [0.0408,0.0450] | No | 0.0430 | [0.0408,0.0451] | No |
| Degree Level | All | 0.3853 | [0.3322,0.4360] | No | 0.3861 | [0.3322,0.4401] | No | 0.4904 | [0.3313,0.6525] | No |
|  |  |  |  |  |  |  |  |  |  |  |
| ns(Time,4) 1 | ≥ 3.5 vs < 3.5 | 0.3558 | [0.2256,0.4779] | No | 0.2872 | [0.1220,0.4480] | No | 0.3415 | [0.1968,0.4888] | No |
|  | ≥ 3.7 vs < 3.7 |  |  |  | 0.2939 | [0.1511,0.4358] | No |  |  |  |
|  | ≥ 3.8 vs < 3.8 |  |  |  | 0.3600 | [0.2245,0.4972] | No |  |  |  |
|  | ≥ 3.9 vs < 3.9 |  |  |  | 0.4145 | [0.2645,0.5648] | No |  |  |  |
|  | = 4.0 vs < 4.0 |  |  |  | 0.4813 | [0.2621,0.7008] | No |  |  |  |
| ns(Time,4) 2 | ≥ 3.5 vs < 3.5 | 0.2728 | [0.1223,0.4203] | No | 0.3308 | [0.1443,0.5151] | No | 0.3341 | [0.1621,0.5026] | No |
|  | ≥ 3.7 vs < 3.7 |  |  |  | 0.1929 | [0.0279,0.3556] | No |  |  |  |
|  | ≥ 3.8 vs < 3.8 |  |  |  | 0.2011 | [0.0427,0.3575] | No |  |  |  |
|  | ≥ 3.9 vs < 3.9 |  |  |  | 0.3339 | [0.1683,0.4970] | No |  |  |  |
|  | = 4.0 vs < 4.0 |  |  |  | 0.4049 | [0.1932,0.6157] | No |  |  |  |
| ns(Time,4) 3 | ≥ 3.5 vs < 3.5 | 1.0211 | [0.7560,1.2772] | No | 1.0311 | [0.7115,1.3558] | No | 1.1969 | [0.8934,1.5063] | No |
|  | ≥ 3.7 vs < 3.7 |  |  |  | 0.8604 | [0.5714,1.1589] | No |  |  |  |
|  | ≥ 3.8 vs < 3.8 |  |  |  | 0.9091 | [0.6233,1.1944] | No |  |  |  |
|  | ≥ 3.9 vs < 3.9 |  |  |  | 1.0852 | [0.7775,1.3862] | No |  |  |  |
|  | = 4.0 vs < 4.0 |  |  |  | 1.2372 | [0.7930,1.7069] | No |  |  |  |
| ns(Time,4) 4 | ≥ 3.5 vs < 3.5 | 1.2736 | [1.0975,1.4506] | No | 1.1169 | [0.9065,1.3292] | No | 1.4095 | [1.2214,1.6020] | No |
|  | ≥ 3.7 vs < 3.7 |  |  |  | 1.1582 | [0.9733,1.3497] | No |  |  |  |
|  | ≥ 3.8 vs < 3.8 |  |  |  | 1.2006 | [1.0242,1.3840] | No |  |  |  |
|  | ≥ 3.9 vs < 3.9 |  |  |  | 1.3065 | [1.1253,1.4906] | No |  |  |  |
|  | = 4.0 vs < 4.0 |  |  |  | 1.5228 | [1.3068,1.7377] | No |  |  |  |
| ns(Time,4) 1 x Degree Level | All | - | - | - | - | - | - | 0.0607 | [-0.1372,0.2538] | No |
| ns(Time,4) 2 x Degree Level | All | - | - | - | - | - | - | -0.1376 | [-0.3251,0.0467] |  |
| ns(Time,4) 3 x Degree Level | All | - | - | - | - | - | - | -0.4033 | [-0.7849,-0.0192] |  |
| ns(Time,4) 4 x Degree Level | All | - | - | - | - | - | - | -0.2607 | [-0.4590,-0.0676] |  |
|  |  |  |  |  |  |  |  |  |  |  |
| **Thresholds** |  |  |  |  |  |  |  |  | - | - |
| ≥ 3.5 vs < 3.5 | - | -1.9075 | - | - | -1.8930 | - | - | -1.8502 | - | - |
| ≥ 3.7 vs < 3.7 | - | -0.6343 | - | - | -0.6877 | - | - | -0.5766 | - | - |
| ≥ 3.8 vs < 3.8 | - | 0.1211 | - | - | 0.0973 | - | - | 0.1792 | - | - |
| ≥ 3.9 vs < 3.9 | - | 1.1626 | - | - | 1.2161 | - | - | 1.2212 | - | - |
| = 4.0 vs < 4.0 | - | 2.9657 | - | - | 3.0899 | - | - | 3.0248 |  |  |
|  |  |  |  |  |  |  |  |  |  |  |
| **Model Comparison** |  |  |  |  |  |  |  |  |  |  |
|  |  | M1 vs M2 | M1 vs M3 |  | M1 vs M2 | M2 vs M3 |  | M1 vs M3 | M2 versus M3 |  |
| elpd_diff |  | -3.8 | -7.7 |  | 0.0000 | -3.9 |  | 0.0000 | 0.0000 |  |
| se_diff |  | 6.4 | 4.4 |  | 0.0000 | 7.7 |  | 0.0000 | 0.0000 |  |
| *Notes.* - Two sets of models were fitted: a set of models without the time x degree interaction and another model with the interaction term. The purpose was to perform model comparison to see whether there is evidence to support the idea that the magnitude of grade inflation differs by degree level. - As a sensitivity check, Bayesian multilevel ordinal regression models were fitted using the "brms" package in R. 4 chains are used with 2000-2250 warm-up iterations, followed by 3000-3500 iterations. A Bayesian approach as opposed to a frequentist approach was chosen as traditional, frequentist methods can struggle to produce stable results for complex models with many parameters estimated and a Bayesian approach is generally better equipped at handling more complex effects (e.g., random slopes and cross-level interactions). - Estimate is the mean of the posterior distribution for a given parameter and they are expressed in log odds given that a cumulative logit link function was used.  - 95% Crl represents the 95% credibility interval which denotes that there is 95% probability that the true value of the parameter lies within the range.  - Model comparison was performed by comparing examining the leave-one-out cross-validation information criterion for the proportional odds model and the non-proportional odds model. Note that to ensure stable estimates and to prevent the model from being overparameterized, the assumption of proportion odds was only tested with time and no other predictors. elpd represents the difference in expected log predictive density (elpd) between the two models and se represents the difference in standard error. - Results did not show evidence for the violation for the proportional odds assumption for both sets of models. To prevent overparameterization, the PO assumption was only tested for the time variable. Importantly, partial PO model was fitted based on model 3. The category-specific effects of the non-linear time effects were first estimated. Results showed convergence issues and we then simplified the model to only estimate the category-specific effects of linear time effects. Convergence issues were persistent. This suggests that the model might be overparameterized and the more parsimonious model (i.e., Model 3), therefore, we concluded that there is no evidence for the violation for the proportional odds assumption. - Results from model comparison provide some evidence that Model 3 performed better in predictive accuracy compared to Model 1. Although the standard error of the ELPD difference was relatively large, which limits the strength of conclusions that can be drawn, the point estimate still favored the interaction model. Such findings consistent with our results from linear mixed effects model which shows that the effect of time may differ across degree levels. | | | | | | | | | | |
