# Supplementary material for: Graduate grade inflation at a U.S. research-intensive university: A 22-year longitudinal analysis
Source: PLoS One. 2026 Mar 25;21(3):e0341315. doi: 10.1371/journal.pone.0341315 (PMC13016357; doi:10.1371/journal.pone.0341315)
Supplement: S1 File — Table S1a. List of CIP master’s programs included in the current study. Table S1B. List of CIP doctoral programs included in the current study. Table S2a. Results from linear mixed-effects models for master’s programs. Table S2b. Results from linear mixed-effects Models for doctoral programs. Table S3a. Results from Bayesian multilevel ordinal models for master’s programs. Table S3b. Results from Bayesian multilevel ordinal models for doctoral programs. Table S4. Results from Bayesian multilevel ordinal models for both degree levels. (ZIP) [file pone.0341315.s001.zip › Supporting Information/Supporting information - Table S2b.docx]

| **Table S2b. Results from linear mixed-effects models for doctoral programs** | | | | | | | | | |
| --- | --- | --- | --- | --- | --- | --- | --- | --- | --- |
|  | Model 1:  Random Intercepts Model | | | Model 2:  Random Intercepts Model with Linear Time Slope | | | Model 3:  Random Intercepts-Random Slopes Model | | |
| **Fixed Effects** | *b* | *95% CI* | *p* | *b* | *95% CI* | *p* | *b* | *95% CI* | *p* |
| Intercept | 3.7793 | [3.7557,3.803] | <.001 | 3.7260 | [3.6955,3.7564] | <.001 | 3.7214 | [3.6906,3.7521] | <.001 |
| Time | - | - | - | 0.0049 | [0.0038,0.006] | <.001 | 0.0053 | [0.0042,0.0064] | <.001 |
|  |  |  |  |  |  |  |  |  |  |
| **Random Effects** |  |  |  |  |  |  |  |  |  |
| $\sigma^{2}$ | 0.0391 |  |  | 0.0383 |  |  | 0.0378 |  |  |
|  |  |  |  |  |  |  |  |  |  |
| Marginal R^2^/ Conditional R^2^ | 0.00/0.22 |  |  | 0.02/0.24 |  |  | 0.02/0.25 |  |  |
|  |  |  |  |  |  |  |  |  |  |
| **Model Comparison** | - |  |  | $\chi^{2}$(1) = 351.24, *p* <.001 | | | $\chi^{2}$(2) = 126.51, *p* <.001 | | |
|  |  |  |  |  |  |  |  |  |  |
|  |  |  |  |  |  |  |  |  |  |
|  |  |  |  |  |  |  |  |  |  |
| *Notes.* N = 15,701 | | | | | | | | | |

| **Table S2b. (cont'd)** | | | | | | | | | |
| --- | --- | --- | --- | --- | --- | --- | --- | --- | --- |
|  | Model 4:  Random Intercepts-Random Slopes Model | | | Model 5:  Adding demographic variables | | | Model 6:  Adding GRE as Level 1 covariate | | |
| **Fixed Effects** | *b* | *95% CI* | *p* | *b* | *95% CI* | *p* | *b* | *95% CI* | *p* |
| Intercept | 3.7363 | [3.7089,3.7637] | <.001 | 3.7481 | [3.7188,3.7774] | <.001 | 3.7578 | [3.7268,3.7888] | <.001 |
| ns(Time,4) 1 | 0.0506 | [0.0286,0.0726] | <.001 | 0.0519 | [0.0298,0.0739] | <.001 | 0.0495 | [0.027,0.072] | <.001 |
| ns(Time,4) 2 | 0.0428 | [0.0221,0.0635] | <.001 | 0.0463 | [0.025,0.0677] | <.001 | 0.0347 | [0.0137,0.0557] | .002 |
| ns(Time,4) 3 | 0.0985 | [0.0618,0.1353] | <.001 | 0.1026 | [0.0658,0.1394] | <.001 | 0.0934 | [0.058,0.1287] | <.001 |
| ns(Time,4) 4 | 0.1215 | [0.0976,0.1455] | <.001 | 0.1260 | [0.1017,0.1503] | <.001 | 0.1161 | [0.0923,0.1398] | <.001 |
| Sex | - | - | - | -0.0092 | [-0.0175,-0.0009] | .035 | -0.0125 | [-0.021,-0.0041] | .006 |
| Ethnicity (Blacks) | - | - | - | -0.1215 | [-0.1451,-0.0979] | <.001 | -0.0983 | [-0.1221,-0.0746] | <.001 |
| Ethnicity (Hispanics) | - | - | - | -0.0586 | [-0.0805,-0.0367] | <.001 | -0.0431 | [-0.0641,-0.0221] | <.001 |
| Ethnicity (Asians) | - | - | - | -0.0120 | [-0.0329,0.0088] | .265 | -0.0173 | [-0.0381,0.0034] | .110 |
| Ethnicity (Others) | - | - | - | -0.1069 | [-0.1391,-0.0747] | <.001 | -0.0843 | [-0.1175,-0.051] | <.001 |
| Ethnicity (Not Specified) | - | - | - | 0.0062 | [-0.0142,0.0267] | .554 | 0.0042 | [-0.0159,0.0243] | .686 |
| GRE Missingness Indicator | - | - | - | - | - | - | 0.0008 | [-0.013,0.0147] | .907 |
| GRE Total Score | - | - | - | - | - | - | 0.0034 | [0.0029,0.004] | <.001 |
|  |  |  |  |  |  |  |  |  |  |
| **Random Effects** |  |  |  |  |  |  |  |  |  |
| $\sigma^{2}$ | 0.0377 |  |  | 0.0370 |  |  | 0.0362 |  |  |
|  |  |  |  |  |  |  |  |  |  |
| Marginal R^2^/ Conditional R^2^ | 0.02/0.33 |  |  | 0.03/0.33 |  |  | 0.05/0.35 |  |  |
|  |  |  |  |  |  |  |  |  |  |
| **Model Comparison** | $\chi^{2}$(3) = 30.79, *p* <.001 | | | $\chi^{2}$(6) = 261.84, *p* <.001 | | | $\chi^{2}$(2) = 373.72, *p* <.001 | | |
| *Notes. N* = 15,701; ns refers to natural splines which were used to model the non-linear effects of time; GRE total score was mean-centered to facilitate intercept interpretation; reference group for Ethnicity is Whites and reference group for Sex is Female; missingness indicator (0 = not missing; 1 = missing); degree level (0 = masters; 1 = PhD) | | | | | | | | | |
