# Supplementary material for: Graduate grade inflation at a U.S. research-intensive university: A 22-year longitudinal analysis
Source: PLoS One. 2026 Mar 25;21(3):e0341315. doi: 10.1371/journal.pone.0341315 (PMC13016357; doi:10.1371/journal.pone.0341315)
Supplement: S1 File — Table S1a. List of CIP master’s programs included in the current study. Table S1B. List of CIP doctoral programs included in the current study. Table S2a. Results from linear mixed-effects models for master’s programs. Table S2b. Results from linear mixed-effects Models for doctoral programs. Table S3a. Results from Bayesian multilevel ordinal models for master’s programs. Table S3b. Results from Bayesian multilevel ordinal models for doctoral programs. Table S4. Results from Bayesian multilevel ordinal models for both degree levels. (ZIP) [file pone.0341315.s001.zip › Supporting Information/Supporting Information - Table S1b.docx]

**Table S1b. List of CIP doctoral programs included in the current study**

| # | **CIP program** |
| --- | --- |
| 1 | Aerospace, Aeronautical, and Astronautical/Space Engineering, General |
| 2 | Agricultural Engineering |
| 3 | American/United States Studies/Civilization |
| 4 | Animal Sciences, General |
| 5 | Anthropology, General |
| 6 | Art History, Criticism and Conservation |
| 7 | Astrophysics |
| 8 | Atomic/Molecular Physics |
| 9 | Biochemistry and Molecular Biology |
| 10 | Bioengineering and Biomedical Engineering |
| 11 | Bioinformatics |
| 12 | Biological and Biomedical Sciences, Other |
| 13 | Biology/Biological Sciences, General |
| 14 | Biomathematics, Bioinformatics, and Computational Biology, Other |
| 15 | Biostatistics |
| 16 | Botany/Plant Biology, Other |
| 17 | Business Administration and Management, General |
| 18 | Chemical Engineering |
| 19 | Chemistry, General |
| 20 | Civil Engineering, General |
| 21 | Classics and Classical Languages, Literatures, and Linguistics, General |
| 22 | Communication, Journalism, and Related Programs, Other |
| 23 | Computer Science |
| 24 | Curriculum and Instruction |
| 25 | Design and Applied Arts, Other |
| 26 | Developmental and Child Psychology |
| 27 | Drama and Dramatics/Theatre Arts, General |
| 28 | Ecology |
| 29 | Econometrics and Quantitative Economics |
| 30 | Educational Leadership and Administration, General |
| 31 | Educational Psychology |
| 32 | Electrical and Electronics Engineering |
| 33 | English Language and Literature, General |
| 34 | Entomology |
| 35 | Environmental Health |
| 36 | Epidemiology |
| 37 | Exercise Physiology and Kinesiology |
| 38 | Family and Consumer Economics and Related Services, Other |
| 39 | Food Science |
| 40 | French Language and Literature |
| 41 | Geography |
| 42 | Geology/Earth Science, General |
| 43 | Health and Medical Administrative Services, Other |
| 44 | History and Philosophy of Science and Technology |
| 45 | History, General |
| 46 | Human Computer Interaction |
| 47 | Hydrology and Water Resources Science |
| 48 | Industrial Engineering |
| 49 | Mass Communication/Media Studies |
| 50 | Materials Engineering |
| 51 | Mathematics, General |
| 52 | Mechanical Engineering |
| 53 | Medicinal and Pharmaceutical Chemistry |
| 54 | Microbiology and Immunology |
| 55 | Molecular Biology |
| 56 | Molecular Pharmacology |
| 57 | Multi-/Interdisciplinary Studies, Other |
| 58 | Music, General |
| 59 | Natural Resources Conservation and Research, Other |
| 60 | Natural Resources/Conservation, General |
| 61 | Neuroscience |
| 62 | Nursing Administration |
| 63 | Nutrition Sciences |
| 64 | Pharmaceutics and Drug Design |
| 65 | Pharmacology |
| 66 | Philosophy |
| 67 | Physics, General |
| 68 | Plant Pathology/Phytopathology |
| 69 | Plant Sciences, General |
| 70 | Political Science and Government, General |
| 71 | Public Administration |
| 72 | Rehabilitation Science |
| 73 | Research and Experimental Psychology, Other |
| 74 | Rhetoric and Composition |
| 75 | Romance Languages, Literatures, and Linguistics, Other |
| 76 | Social Work |
| 77 | Sociology, General |
| 78 | Statistics, General |
